# Risk assessment of human exposure to airborne pesticides in rural greenhouses

**DOI:** 10.1038/s41598-023-32458-y

**Published:** 2023-03-29

**Authors:** Yuzhao Hu, Shuai Wu, Wei Lyu, Jun Ning, Dongmei She

**Affiliations:** 1 State Key Laboratory for Biology of Plant Diseases and Insect Pests, Institute of Plant Protection, Chinese Academy of Agricultural Sciences, Beijing, 100193 China; 2 Hebei Science and Technology Innovation Service Center, Hebei, 050035 China

**Keywords:** Environmental chemistry, Environmental impact, Risk factors

## Abstract

In comparison to an open field, greenhouses utilize much more pesticides. The non-occupational exposure risk caused by pesticide drift is unknown. In this study, within 8 months (from March 2018 to October 2018), air samples were collected from indoor and outdoor houses and public areas near greenhouses in vegetable growing areas (eggplant, leek, garlic, etc.), and qualitative and quantitative analyses of pesticides were carried out. Using a 95% confidence interval, six pesticides (acetamiprid, difenoconazole, thiazophos, isoprocarb, malathion, and pyridaben) were detected. The results of the safety assessment showed that the non-cancer exposure risk of single pesticides for all residents in the agricultural areas was within the acceptable range, and the excess lifetime cancer risk of all residents inhaling difenoconazole exceeded 1E−6, and the agricultural region urgently needs increased cancer regulatory scrutiny. But combined toxicity of six pesticides not evaluated due to lack of suitable data. Comparison with open field scenes, the results show that pesticide levels to airborne are lower in greenhouse regions.

## Introduction

Greenhouses are convenient for planting crops in various climatic conditions and use relatively less space and lower input to improve the unit land output and land utilization rate. However, warm and humid conditions in greenhouses also increase the occurrence of pests, causing a surge in pesticide use^1,2^. A survey conducted in Hebei Province revealed that the amount of pesticides per hectare in greenhouses was almost four times higher than that in open fields^3^.

Dispersed gases and dust are carried by air into the surrounding environment and residential buildings, resulting in nonoccupational exposure. Chemicals in the air can be ingested, inhaled, or absorbed through the skin^4^. It is particularly important to measure pesticides in the air. Given the ubiquitous pollution of pesticides in the air and the fact that people spend most of their time at home, the exposure of dwellings to indoor and outdoor environments is particularly worrying^5^.

Due to their unique labor characteristics, Chinese agricultural practitioners' safety demands have been examined due to the lack of pertinent data. In 2017, China's total greenhouse area was 2,048,518.61 hectares, of which Shandong's greenhouse area was 292,528.83 hectares, ranking second in the country^6^.

In recent studies on pesticide residues in food and environmental samples, not only the definition of the residue and its comparison with the MRL (Maximum Residue Limit) have been made, but also the risk assessments of consumers' exposure to residues^7–10^. Josephine^11^ conducted a newly built residential pesticide exposure study and detected pyrethroid pesticides at exposure levels of 185 ng/g. Ward et al.^12^ evaluated indoor glyphosate exposure and found that the potential exposure of young children was high. Yoshida et al.^13^ detected diazinon at an exposure level of 0.067 mg/kg/day in a study of indoor children exposed to organophosphate pesticides.

To date, neither the release of pesticides outside sheds nor reports of non-occupational exposure to pesticides have been examined or documented. To evaluate the risk of farmers inhaling pesticides daily, we selected a typical Shandong vegetable greenhouse planting area.

## Materials and methods

### Pesticide selection and site characterization

Zhanglaozhuang (36 29′ 38″ N, 116 13′ 01″ E), a typical vegetable cropping area in Shandong, China, located in East China, was selected. This area is only used for growing vegetables, predominantly leeks, garlic, and eggplants. We investigated crop cultivation and pesticide use in this agricultural area. The types of vegetables planted in winter, the application of pesticides and the working methods of residents in this area are not different from those in other seasons. Therefore, we did not sample in winter. And it has been confirmed that most of pesticides almost have no significant season-change. Six typical vegetable growers were selected to investigate pesticides used in spring, summer, and autumn (March to October). Fourteen species of CUPs (Current use pesticides) were investigated and samples were collected monthly. Pesticides used by residents in this agricultural area were phoxim, acetamiprid, abamectin, pyridaben, thiazophos, Beauveria bassiana, malathion, isoprocarb, mushroom proteoglycan, Propineb, prochloraz, difenoconazole, chlorothalonil, and propamocarb hydrochloride.

### Sampling plan

In this experiment, a polyurethane foam sampler (PUF-PAS) developed by Jenna et al.^14^, was used for passive air sampling. This can passively adsorb pesticides in the air while collecting dust in the air, is not significantly affected by human factors in the sampling process, and can be deployed in remote areas without a power supply. At the same time as passive sampling, a deposition plate and active sampling devices (XAD-2 resin is selected as the adsorption material) were deployed to verify the correctness of the sampling method.

When investigating the indoor environment, we mainly consider factors such as pesticide storage locations (including spraying equipment), residents' living habits (such as the time and frequency of staying in a certain indoor area), indoor layout, and try to avoid interference from irrelevant factors. After the first application, the PUF (140 mm × 13.5 mm) was deployed inside and outside seven selected residential buildings in agricultural areas. Indoor samples were placed on 1 m-high tables (human breathing areas), outdoor samples were placed on 1.5 m-high poles, and additional samples were placed in personnel gathering areas to avoid as much contact from other surfaces as possible, and information such as temperature, wind speed, and humidity were recorded at the same time. Samples were collected at the beginning of the month and collected at the end of the month. According to the pesticide application situation of farmers, 199 PUF samples were collected from March to October in spring, summer, and autumn. Each sample contained two replications, and the samples were stored in a refrigerator at − 20 °C, away from light.

### Sample analysis

Soxhlet extraction was used to process the collected PUF. The extraction solvent (100 ml ethyl acetate) and extraction time (8 h) were selected in advance. After Soxhlet extraction, the solution was concentrated by rotary evaporation, whirled, centrifuged, purified to 1.5 ml, and injected into the 1.5 ml chromatography vials. Qualitative and quantitative analyses of the samples were carried out using GC–MS in SIM mode, and the external standard method was used for quantitative analysis. The quality of the samples was controlled through addition and recovery experiments, five different spiking levels (50 ppb, 100 ppb, 1 ppm, 10 ppm, 50 ppm, n = 3) were used with a method average recovery rate of 89% (RSD 1.68% ~ 7.55%). Fifteen pesticides were separated and identified using a GCMS-QP2020 (SHIMADZU) mass spectrometer. The column used was Rtx-5 ms (30 m × 0.25 mm × 0.25 μm). The temperature gradient was 50–160 °C, 30 °C/min; 160–205 °C, 15 °C/min; 205–300 °C, 5 °C/min. The split injection, split ratio was 10:1, inlet temperature 270 °C, and injection volume 2 μL.

### Methods

The HQ (Hazard quotient) of the inhalation pathway was calculated using the following formula (Eq. 1)^15^:

1 $${\text{HQ}} = {\text{EC}}/\left( {{\text{Toxicity}}\;{\text{Value}}^{1} \times 1000\,\upmu {\text{g/mg}}} \right)$$

where HQ (no unit) is the danger quotient and toxicity values (mg/m^3^) are inhalation toxicity values (e.g., RfC) appropriate for exposure scenarios (acute, subchronic, or chronic). The Toxicity Value used in this experiment is RfC (Reference Concentration).

Cancer risk assessment also refers to the Exposure Factor Manual proposed by USEPA^16^ and Industry standard^17^. ADAF (Age Dependent Adjustment Factor) is used to calculate or estimate risks associated with early life exposure if specific chemical data on susceptibility to early life exposure are not available^18^. The following formula was used to calculate the lifetime cancer risk (Eq. 2)^15^.

2 $${\text{Risk}} = \left( {{\text{IUR}}_{{\text{U}}} {\text{EC}}_{ < 2} \times {\text{ADAF}}_{ < 2} } \right) + \left( {{\text{IUR}} \times {\text{EC}}_{2 - 16} \times {\text{ADAF}}_{2 - 16} } \right) + \left( {{\text{IUR}} \times {\text{EC}}_{ > 16} } \right)$$

where EC (μg/m^3^) is the exposure concentration and IUR (μg/m^3^)^−1^ is the unit risk of inhalation.

### Ethics approval

This is an observational study. It is confirmed that no ethical approval is required.

## Results

### Non-cancer risk assessment

**Figure 1:**
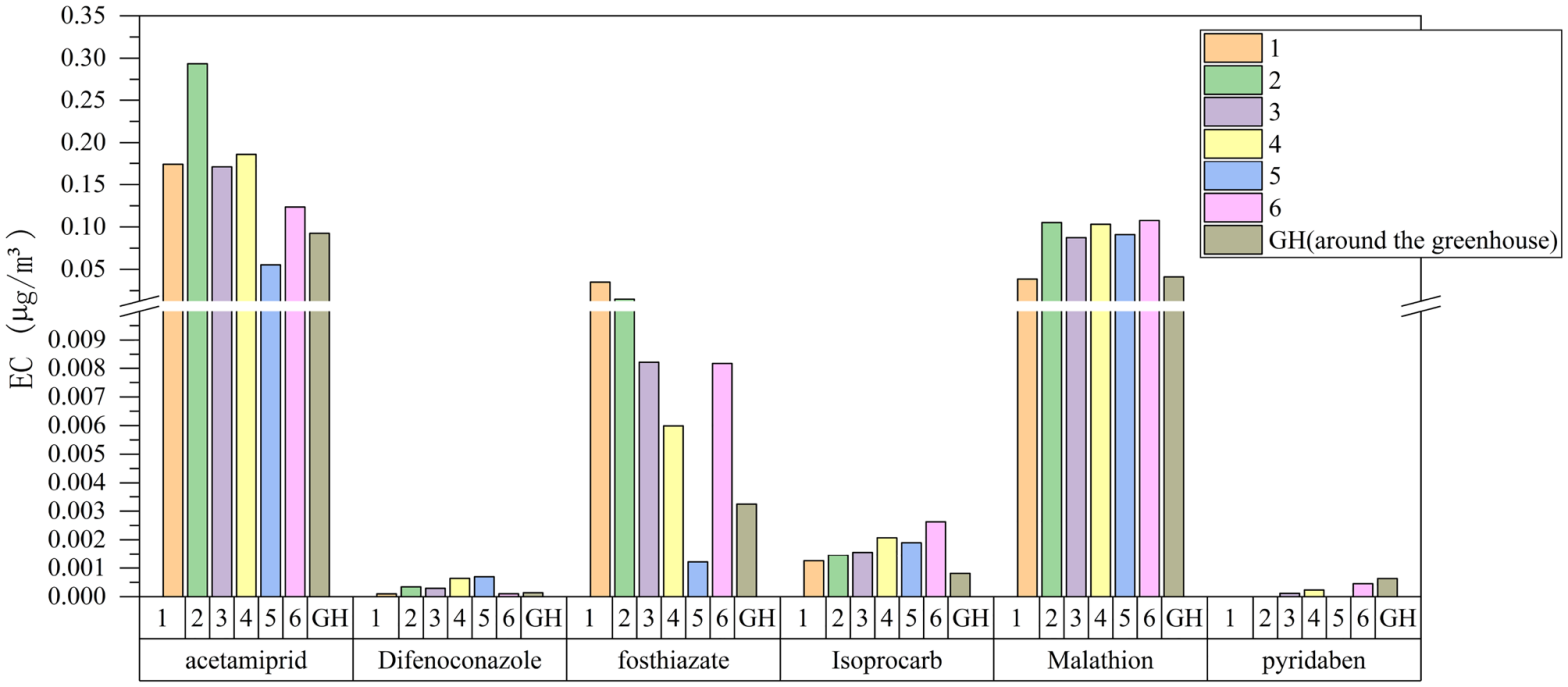
Average EC of 7 households in agricultural areas.

**Figure 2:**
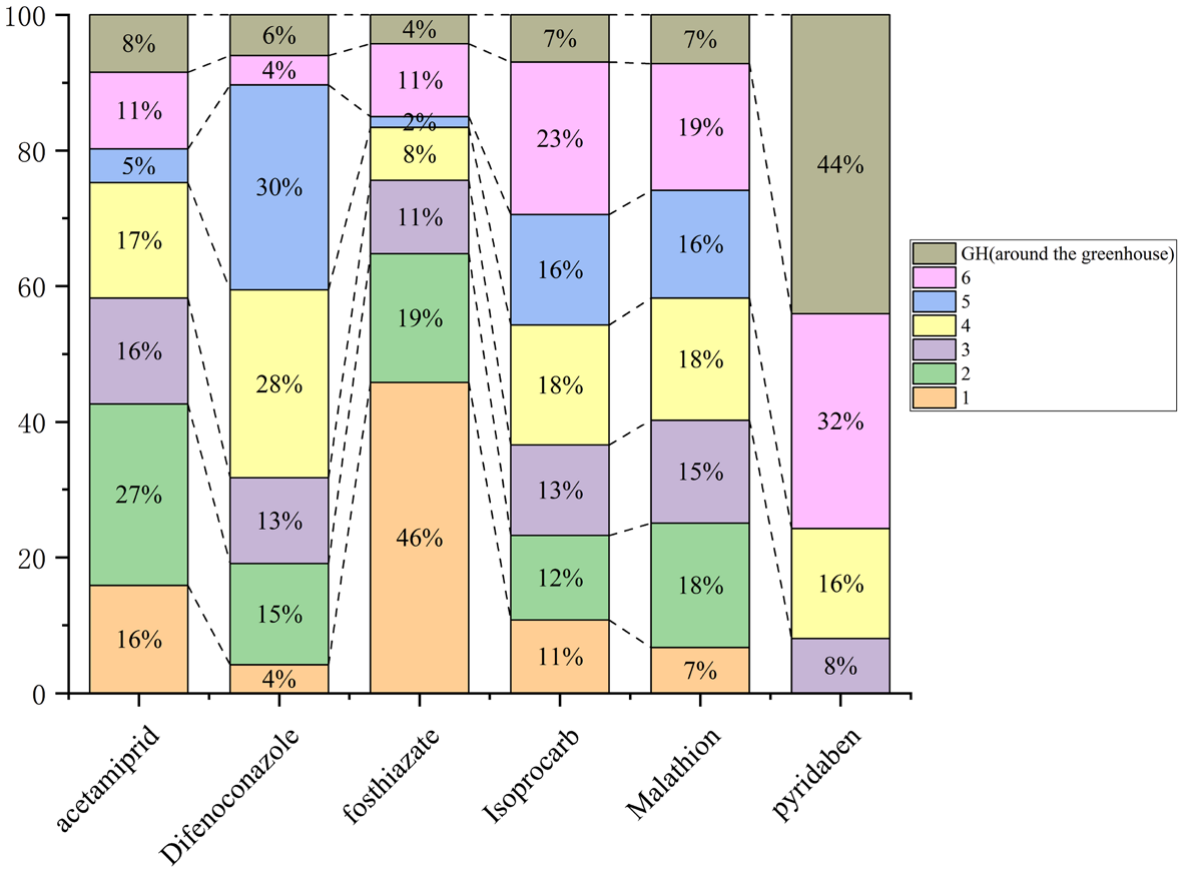
Average EC percentage per household for each pesticide.

Probabilistic estimates of non-cancer risk in six households and one shed (GH, around the greenhouse) in an agricultural community showed that six of the fourteen pesticides applied were acetamiprid, difenoconazole, thiazophos, isoprocarb, malathion, and pyridaben (Figs. 1, 2). Different dosimetric adjustment factors (DAFs) were used for each pesticide exposure scenario and the mean CA (Contaminant concentration in air) was selected. Factors related to early life exposure susceptibilities, such as those in infants and children, were also considered. According to the living habits of residents surveyed at the initial stage of the experiment, the indoor activity time of the residents was 10 h and the outdoor activity time was 4 h. Figure 1 shows the average Exposure Concentration (EC) of the seven households in the agricultural areas.

The calculation results used a 95% confidence interval and the EC of acetamiprid was generally higher than that of pyridaben. The EC of pyridaben was the lowest, and was not detected in residential buildings No. 1, No. 2, and No. 5. The EC of six households and the second household in the shed area were generally the highest, which may be related to factors such as the distance between the residence and the application point.

**Figure 3:**
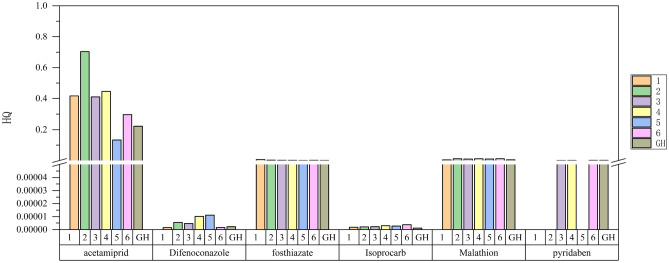
Lifetime non-cancer risk of 7 households exposed to a certain pesticide alone in agricultural areas.

Figure 3 lists the lifetime non-cancer risks of seven households exposed only to a certain pesticide in agricultural areas.

After a risk assessment of chronic pesticide inhalation in seven households in vegetable-growing areas, it was found that the exposure risks of all detected pesticides were acceptable. The acetamiprid exposure risk in the second household was the highest, and HQ is 7.04E−01 < 1 (Fig. 3). This means that, in terms of exposure to a single pesticide, residents in agricultural areas are less likely to become ill.

### Cancer risk assessment

**Figure 4:**
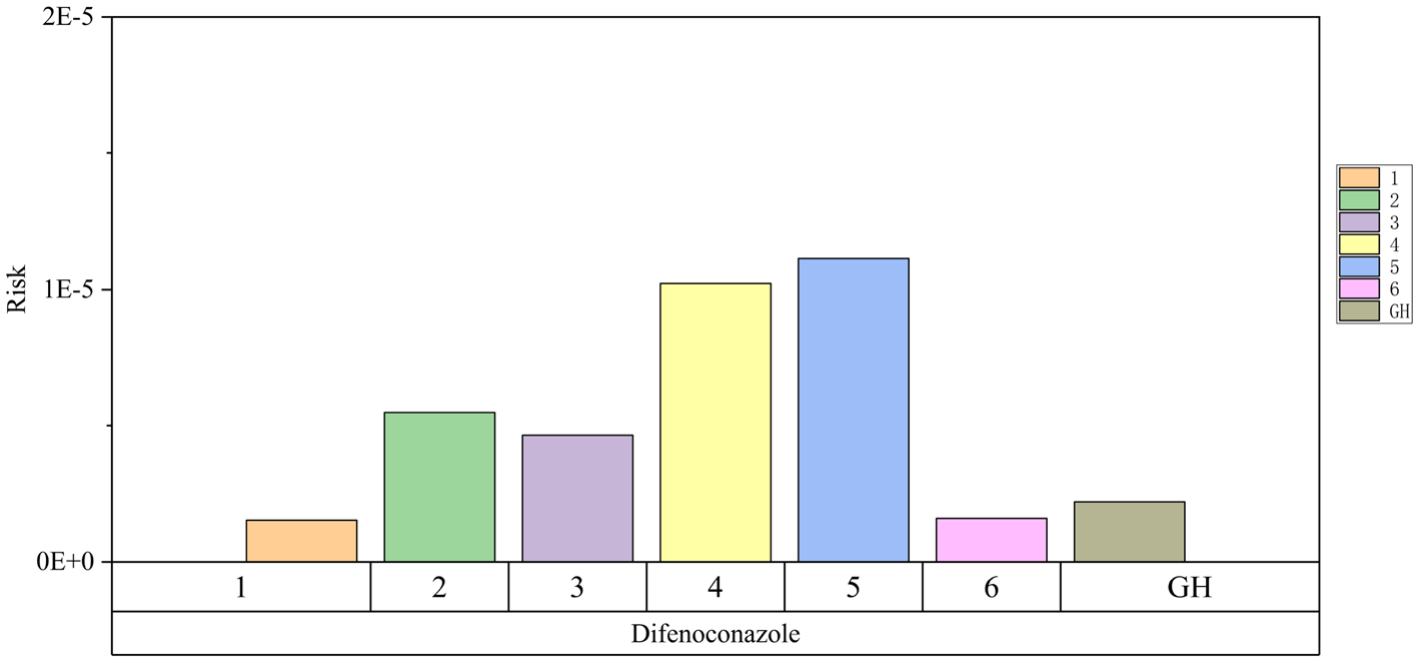
Cancer exposure risk of acetochlor and pendimethalin.

The cancer inhalation risk of the pesticides on the list was evaluated using the classification manual for pesticides and cancer issued by the EPA (U.S. Environmental Protection Agency)^19^. EPA's current process of estimating cancer risk is based on the unit risk estimate (URE) for inhalation. The estimated lifetime cancer risk associated with difenoconazole, a possible human carcinogen, is shown in Fig. 4.

The liver is the main target organ for difenoconazole. Adverse effects of difenoconazole on the liver include hypertrophy, vacuolation, and hepatocytes^20^. The excess lifetime cancer risk in each household and around the greenhouse exceeded 1.0E−6 (Fig. 4). Excess lifetime cancer risk in the range of 1.0E−6 to 1.0E−4 is considered to be used for regulatory purposes to protect human health; therefore, it is urgent to strengthen the regulatory review of cancer risk^21^.

## Discussion

In the inhalation risk assessment, all six pesticides detected were currently used pesticides (CUPs). In the collected air samples, the pesticide with the lowest exposure was difenoconazole, and the pesticide with the highest exposure was acetamiprid. The overall exposure range was 1.29E−05 ~ 1.36E−02 μg/m^3^. Gibbs's^22^ study found similar results. The exposure results of six households show that the concentration of pesticides indoors is generally higher than that outdoors. This may be related to the low frequency of indoor ventilation. In addition, indoor items have an adsorption effect on pesticides. At present, there is no research on the risk assessment of pesticide inhalation exposure for residents in agricultural areas in China. In the absence of reference data, we believe that this may be related to the weak awareness of hygiene of residents related. The risk characterization results showed that the highest excess lifetime cancer risk of difenoconazole was 7.48E−05. This is at a relatively severe level in similar studies^23,24^. Considering the effects of skin pathways and dietary pathways, residents of this agricultural area may actually suffer from greater cancer and non-cancer risks.

As a typical vegetable-growing area in China, Zhang Laozhuang's evaluation results also represent the current status of pesticide use in the vast vegetable-growing areas with similar environmental factors and farmers' labor patterns of China. China has the highest pesticide production and use^25^. During the 2-year risk assessment study, six of the 14 pesticides investigated were detected, which also confirms the heavy use of pesticides in the region. Compared with the open-air field, the pesticide dosage per unit area of the greenhouse is about 3–4 times higher than that of the open-air field^3^. Comparing the experimental results with another study on the risk assessment of pesticide inhalation exposure of residents in grain-growing areas, the cancer and non-cancer risks of residents in vegetable-growing areas were significantly lower than those in grain-growing areas. Considering the closed nature of greenhouses with limited ventilation systems or no ventilation systems, most pesticides are still present in greenhouses even after farmers apply pesticides. This means that the greenhouse has a good limit on pesticide drift. The non-occupational exposure routes of residents around greenhouses are safer, and the occupational exposure risks of pesticides in the corresponding farmers' application processes may be higher.

This experiment refers to the evaluation method recommended by the EPA IRIS and selects the reference value based on the inhalation pathway to ensure the credibility of the experimental results to the greatest extent possible.

It has been reported that when various pesticides are mixed, harm to organisms increases greatly. Mixtures of globally common pesticides can cause up to 99% mortality in larval amphibians, but this effect has not been completely explained by individual pesticide effects^26^. The above assessment is based on the conclusions obtained from the evaluation of a certain exposure pesticide alone, but the actual exposure is that multiple pesticides enter the human body at the same time. Residents of agricultural regions may be at an unacceptable risk of inhalation exposure. Further studies are required to determine the actual toxicity of the combined pesticides in humans.

This experiment preliminarily evaluated the safety of non-occupational inhalation exposure for residents in typical vegetable greenhouse agricultural areas in China, filling the gap in the study of inhalation exposure in agricultural areas in China, Provide a reference for the formulation of policies on pesticide management, drug types and doses, and air pollutants in China.

## Conclusion

In summary, the risk assessment of home exposure to the six pesticides in spring, summer, and autumn was carried out in six households and greenhouses in typical vegetable-growing areas of Shandong Province. The average EC of acetamiprid was the highest. The non-cancer risk assessment for all pesticides did not exceed this limit (HQ < 1). The excess lifetime cancer risk of all residents inhaling difenoconazole exceeded 1E−6, and the agricultural region urgently needs increased cancer regulatory scrutiny. Our results show that the sealing effect of the greenhouse had a limiting effect on pesticide drift.

## Data Availability

Datasets are available from the corresponding author on reasonable request.
